# {6,6′-Dimeth­oxy-2,2′-[cyclo­hexane-1,2-diylbis(nitrilo­methanylyl­idene)]diphenolato}copper(II) monohydrate

**DOI:** 10.1107/S1600536812044625

**Published:** 2012-11-10

**Authors:** Chunmei Hu, Shunsheng Zhao, Xingqiang Lü, Rong Lu

**Affiliations:** a College of Chemical Engineering, Northwest University, Xi’an 710069, Shaanxi, People’s Republic of China; b College of Chemistry and Chemical Engineering, Xi’an University of Science and Technology, Xi’an 710054, Shaanxi, People’s Republic of China

## Abstract

In the title compound, [Cu(C_22_H_24_N_2_O_4_)]·H_2_O, the Cu^II^ atom is four-coordinated in a distorted planar geometry with a mean deviation of 0.1164 (2) Å for the plane generated by the ligating atoms of the salen-type Schiff base ligand. In the crystal, O(water)—H⋯O and C—H⋯O hydrogen bonds form a three-dimensional-network.

## Related literature

For the synthetic method, see: Marinovich *et al.* (1999 ▶). For related structures, see: Tang (2009 ▶); Ji & Lu (2010 ▶).
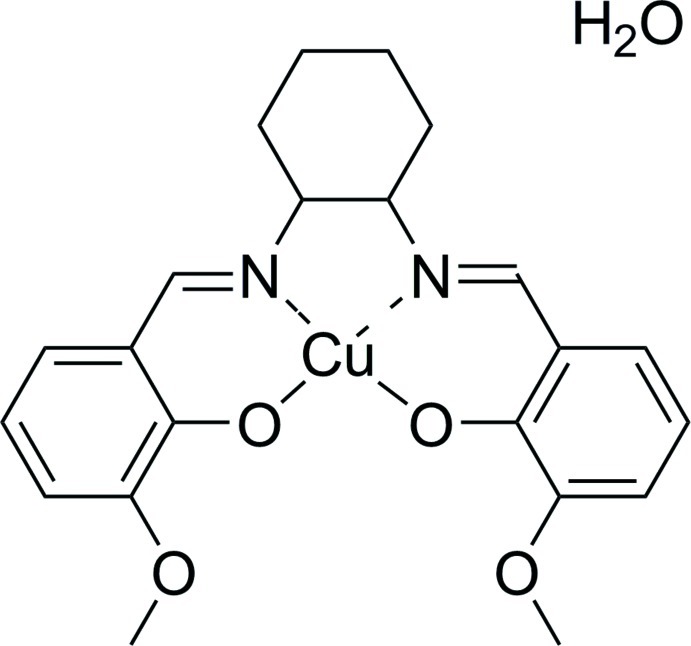


## Experimental

### 

#### Crystal data

[Cu(C_22_H_24_N_2_O_4_)]·H_2_O
*M* *_r_* = 462.00
Monoclinic, 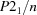

*a* = 11.2116 (13) Å
*b* = 10.5256 (12) Å
*c* = 18.171 (7) Å
β = 106.185 (2)°
*V* = 2059.4 (9) Å^3^
*Z* = 4
Mo *K*α radiation
μ = 1.10 mm^−1^
*T* = 296 K
0.38 × 0.24 × 0.21 mm

#### Data collection

Bruker SMART 1K CCD area-detector diffractometer
Absorption correction: multi-scan (*SADABS*; Sheldrick, 2004 ▶) *T*_min_ = 0.736, *T*_max_ = 0.794
10265 measured reflections
3670 independent reflections
2859 reflections with *I* > 2σ(*I*)
*R*_int_ = 0.048

#### Refinement

*R*[*F* ^2^ > 2σ(*F* ^2^)] = 0.050
*wR*(*F* ^2^) = 0.105
*S* = 1.05
3670 reflections
279 parameters
3 restraints
H-atom parameters constrained
Δρ_max_ = 0.30 e Å^−3^
Δρ_min_ = −0.30 e Å^−3^

### 

Data collection: *SMART* (Bruker, 2001 ▶); cell refinement: *SAINT* (Bruker, 2001 ▶); data reduction: *SAINT*; program(s) used to solve structure: *SHELXS97* (Sheldrick, 2008 ▶); program(s) used to refine structure: *SHELXL97* (Sheldrick, 2008 ▶); molecular graphics: *SHELXTL* (Sheldrick, 2008 ▶); software used to prepare material for publication: *SHELXTL* and local programs.

## Supplementary Material

Crystal structure: contains datablock(s) I, global. DOI: 10.1107/S1600536812044625/qm2087sup1.cif

Structure factors: contains datablock(s) I. DOI: 10.1107/S1600536812044625/qm2087Isup2.hkl

Additional supplementary materials:  crystallographic information; 3D view; checkCIF report

## Figures and Tables

**Table 1:** Hydrogen-bond geometry (Å, °)

*D*—H⋯*A*	*D*—H	H⋯*A*	*D*⋯*A*	*D*—H⋯*A*
O5—H*W*1⋯O1^i^	0.85	2.24	2.971 (5)	146
O5—H*W*1⋯O2^i^	0.85	2.48	3.161 (4)	138
C8—H8*A*⋯O3^ii^	0.93	2.44	3.352 (4)	166
C9—H9*A*⋯O2^ii^	0.98	2.65	3.579 (5)	159

Symmetry codes: (i) 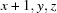; (ii) 
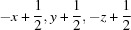
.

**Fig. 1.**
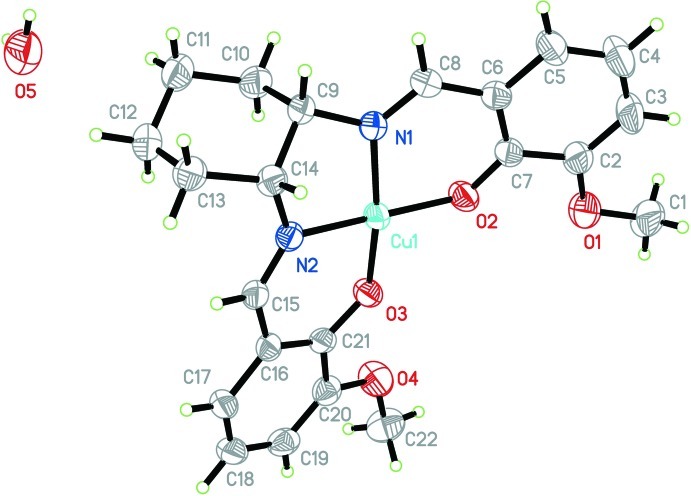
The molecular structure of (I), with atom labels and 50% probability displacement ellipsoids for non-H atoms.

**Fig. 2.**
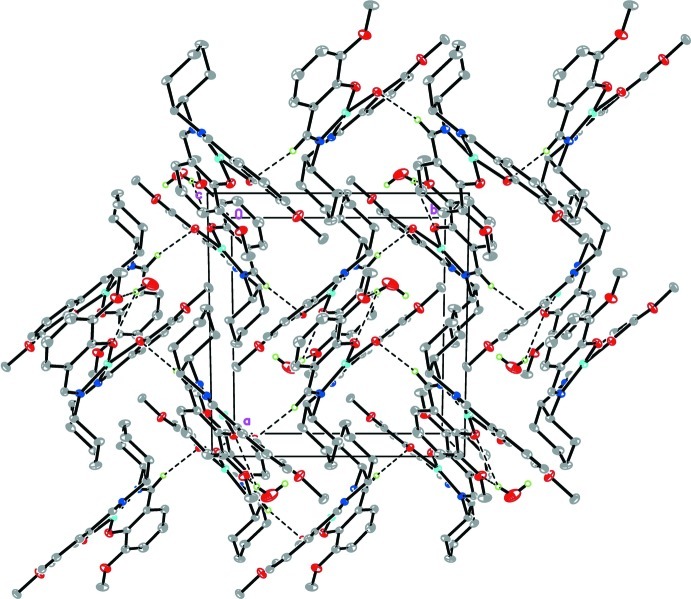
The packing diagram of (I), viewing from c axis direction.

## supplementary crystallographic information

### Comment

Salen Schiff-bases and their metal complexes are of interest due to their biological activity, as well as their optical, catalytic, chromophoric, thermochromic and photochromic properties. Here we report the crystal structure of a salen-type Schiff-base copper complex, Fig. 1. The title compound crystallized with one independent molecule and one water molecule in the asymmetric unit. Each of the Cu atoms is in an almost planar coordination geometry and is close to the plane defined by the four ligating atoms (N1, N2, O2, O3) of the Schiff-base ligand. Fig. 2 shows the packing diagram along the crystallographic *c* axis.

### Experimental

The compound was prepared according to previous reported method of Marinovich (1999). Crystals suitable for the X-ray diffraction study were obtained upon recrystallization from *N,N*-dimethylformamide and water.

### Refinement

H atoms were positioned geometrically and refined using a riding model with C—H = 0.95–0.99 Å and with *U*_iso_(H) = 1.2 times *U*_eq_(C).Water H atoms were located in a difference-Fourier synthesis and refined with constraint O—H = 0.82 Å, H—H distance 1.35 Å and *U*_iso_(H) = 1.5Ueq(O).

### Crystal data

**Table:**

[Cu(C_22_H_24_N_2_O_4_)]·H_2_O	*F*(000) = 964
*M**_r_* = 462.00	*D*_x_ = 1.490 Mg m^−^^3^
Monoclinic, *P*2_1_/*n*	Mo *K*α radiation, λ = 0.71073 Å
Hall symbol: -P 2yn	Cell parameters from 5789 reflections
*a* = 11.2116 (13) Å	θ = 1.9–25.3°
*b* = 10.5256 (12) Å	µ = 1.10 mm^−^^1^
*c* = 18.171 (7) Å	*T* = 296 K
β = 106.185 (2)°	Block, dark green
*V* = 2059.4 (9) Å^3^	0.38 × 0.24 × 0.21 mm
*Z* = 4	

### Data collection

**Table:**

Bruker SMART 1K CCD area-detector diffractometer	3670 independent reflections
Radiation source: fine-focus sealed tube	2859 reflections with *I* > 2σ(*I*)
Graphite monochromator	*R*_int_ = 0.048
thin–slice ω scans	θ_max_ = 25.1°, θ_min_ = 2.3°
Absorption correction: multi-scan (*SADABS*; Sheldrick, 2004)	*h* = −13→11
*T*_min_ = 0.736, *T*_max_ = 0.794	*k* = −12→8
10265 measured reflections	*l* = −21→21

### Refinement

**Table:**

Refinement on *F*^2^	Primary atom site location: structure-invariant direct methods
Least-squares matrix: full	Secondary atom site location: difference Fourier map
*R*[*F*^2^ > 2σ(*F*^2^)] = 0.050	Hydrogen site location: inferred from neighbouring sites
*wR*(*F*^2^) = 0.105	H-atom parameters constrained
*S* = 1.05	*w* = 1/[σ^2^(*F*_o_^2^) + (0.0397*P*)^2^ + 0.7087*P*] where *P* = (*F*_o_^2^ + 2*F*_c_^2^)/3
3670 reflections	(Δ/σ)_max_ < 0.001
279 parameters	Δρ_max_ = 0.30 e Å^−^^3^
3 restraints	Δρ_min_ = −0.30 e Å^−^^3^

### Special details

**Table:**

Geometry. All e.s.d.'s (except the e.s.d. in the dihedral angle between two l.s. planes) are estimated using the full covariance matrix. The cell e.s.d.'s are taken into account individually in the estimation of e.s.d.'s in distances, angles and torsion angles; correlations between e.s.d.'s in cell parameters are only used when they are defined by crystal symmetry. An approximate (isotropic) treatment of cell e.s.d.'s is used for estimating e.s.d.'s involving l.s. planes.
Refinement. Refinement of *F*^2^ against ALL reflections. The weighted *R*-factor *wR* and goodness of fit *S* are based on *F*^2^, conventional *R*-factors *R* are based on *F*, with *F* set to zero for negative *F*^2^. The threshold expression of *F*^2^ > σ(*F*^2^) is used only for calculating *R*-factors(gt) *etc*. and is not relevant to the choice of reflections for refinement. *R*-factors based on *F*^2^ are statistically about twice as large as those based on *F*, and *R*- factors based on ALL data will be even larger.

### Fractional atomic coordinates and isotropic or equivalent isotropic displacement parameters (Å^2^)

**Table:**

	*x*	*y*	*z*	*U*_iso_*/*U*_eq_	
Cu1	0.14977 (4)	0.96825 (4)	0.16126 (2)	0.03146 (16)	
N2	0.2322 (3)	1.0281 (3)	0.08566 (17)	0.0313 (7)	
O3	0.0574 (2)	0.8409 (2)	0.09541 (14)	0.0360 (6)	
O2	0.0640 (2)	0.9319 (2)	0.23616 (14)	0.0395 (6)	
N1	0.2692 (3)	1.0751 (3)	0.23096 (17)	0.0339 (7)	
C15	0.2008 (3)	0.9961 (3)	0.0149 (2)	0.0336 (9)	
H15A	0.2363	1.0418	−0.0175	0.040*	
C21	0.0524 (3)	0.8231 (3)	0.0229 (2)	0.0313 (8)	
C8	0.2583 (3)	1.1234 (3)	0.2941 (2)	0.0344 (9)	
H8A	0.3200	1.1787	0.3207	0.041*	
O4	−0.0811 (3)	0.6552 (3)	0.02914 (16)	0.0543 (8)	
C7	0.0660 (3)	1.0045 (3)	0.2949 (2)	0.0354 (9)	
C17	0.0985 (3)	0.8705 (4)	−0.0979 (2)	0.0393 (9)	
H17A	0.1395	0.9201	−0.1255	0.047*	
C14	0.3202 (3)	1.1341 (3)	0.1158 (2)	0.0328 (8)	
H14A	0.2715	1.2126	0.1102	0.039*	
C20	−0.0255 (3)	0.7221 (3)	−0.0167 (2)	0.0379 (9)	
O1	−0.1149 (3)	0.8971 (3)	0.29982 (16)	0.0559 (8)	
C9	0.3749 (3)	1.1096 (4)	0.2019 (2)	0.0363 (9)	
H9A	0.4132	1.1878	0.2270	0.044*	
C6	0.1570 (3)	1.0974 (3)	0.3262 (2)	0.0355 (9)	
C16	0.1160 (3)	0.8963 (3)	−0.0194 (2)	0.0305 (8)	
C2	−0.0302 (4)	0.9896 (4)	0.3321 (2)	0.0437 (10)	
C19	−0.0380 (4)	0.6995 (4)	−0.0931 (2)	0.0422 (10)	
H19A	−0.0881	0.6329	−0.1176	0.051*	
C22	−0.1678 (4)	0.5579 (4)	−0.0063 (3)	0.0573 (12)	
H22A	−0.2006	0.5185	0.0316	0.086*	
H22B	−0.1264	0.4951	−0.0286	0.086*	
H22C	−0.2346	0.5949	−0.0455	0.086*	
C3	−0.0340 (4)	1.0629 (4)	0.3934 (3)	0.0576 (12)	
H3A	−0.0981	1.0516	0.4160	0.069*	
C4	0.0569 (4)	1.1547 (4)	0.4227 (3)	0.0609 (13)	
H4A	0.0534	1.2042	0.4644	0.073*	
C18	0.0231 (4)	0.7747 (4)	−0.1342 (2)	0.0440 (10)	
H18A	0.0125	0.7597	−0.1861	0.053*	
C5	0.1502 (4)	1.1707 (4)	0.3898 (2)	0.0449 (10)	
H5A	0.2111	1.2314	0.4094	0.054*	
C1	−0.2129 (4)	0.8727 (5)	0.3334 (3)	0.0675 (14)	
H1A	−0.2652	0.8066	0.3054	0.101*	
H1B	−0.2610	0.9486	0.3319	0.101*	
H1C	−0.1787	0.8466	0.3857	0.101*	
C10	0.4703 (3)	1.0043 (4)	0.2179 (2)	0.0451 (10)	
H10A	0.4301	0.9246	0.1988	0.054*	
H10B	0.5063	0.9961	0.2728	0.054*	
C12	0.5193 (4)	1.0508 (4)	0.0944 (2)	0.0541 (12)	
H12A	0.5855	1.0723	0.0718	0.065*	
H12B	0.4817	0.9721	0.0713	0.065*	
C11	0.5734 (4)	1.0312 (5)	0.1797 (2)	0.0578 (12)	
H11A	0.6189	1.1066	0.2022	0.069*	
H11B	0.6311	0.9604	0.1886	0.069*	
C13	0.4228 (4)	1.1548 (4)	0.0767 (2)	0.0450 (10)	
H13A	0.3859	1.1590	0.0217	0.054*	
H13B	0.4630	1.2357	0.0932	0.054*	
O5	0.8238 (3)	0.7829 (4)	0.1442 (2)	0.1052 (14)	
HW1	0.8557	0.8378	0.1779	0.158*	
HW2	0.8713	0.7194	0.1555	0.158*	

### Atomic displacement parameters (Å^2^)

**Table:**

	*U*^11^	*U*^22^	*U*^33^	*U*^12^	*U*^13^	*U*^23^
Cu1	0.0307 (3)	0.0376 (3)	0.0278 (3)	−0.0044 (2)	0.01097 (19)	−0.0017 (2)
N2	0.0276 (16)	0.0347 (17)	0.0322 (18)	0.0001 (14)	0.0095 (14)	0.0021 (14)
O3	0.0388 (15)	0.0395 (15)	0.0308 (15)	−0.0073 (11)	0.0113 (12)	−0.0041 (11)
O2	0.0434 (16)	0.0465 (16)	0.0312 (15)	−0.0097 (12)	0.0146 (13)	−0.0041 (12)
N1	0.0298 (17)	0.0430 (18)	0.0304 (18)	−0.0042 (14)	0.0108 (14)	−0.0013 (14)
C15	0.028 (2)	0.044 (2)	0.030 (2)	0.0034 (16)	0.0099 (17)	0.0053 (17)
C21	0.028 (2)	0.034 (2)	0.030 (2)	0.0105 (16)	0.0041 (16)	−0.0012 (16)
C8	0.037 (2)	0.034 (2)	0.030 (2)	−0.0010 (17)	0.0071 (18)	−0.0015 (16)
O4	0.0599 (19)	0.0530 (18)	0.054 (2)	−0.0270 (15)	0.0217 (16)	−0.0120 (15)
C7	0.035 (2)	0.044 (2)	0.027 (2)	0.0064 (17)	0.0082 (17)	0.0058 (17)
C17	0.038 (2)	0.051 (3)	0.030 (2)	0.0096 (19)	0.0106 (18)	−0.0013 (18)
C14	0.032 (2)	0.033 (2)	0.033 (2)	−0.0032 (16)	0.0100 (17)	0.0052 (16)
C20	0.035 (2)	0.035 (2)	0.044 (3)	0.0029 (17)	0.0099 (19)	−0.0040 (19)
O1	0.0469 (18)	0.079 (2)	0.0491 (19)	−0.0161 (16)	0.0258 (15)	0.0005 (16)
C9	0.030 (2)	0.047 (2)	0.035 (2)	−0.0065 (17)	0.0123 (17)	−0.0061 (18)
C6	0.035 (2)	0.041 (2)	0.029 (2)	0.0036 (17)	0.0070 (17)	0.0018 (17)
C16	0.027 (2)	0.034 (2)	0.030 (2)	0.0075 (16)	0.0090 (16)	0.0016 (16)
C2	0.040 (2)	0.057 (3)	0.037 (2)	0.000 (2)	0.016 (2)	0.005 (2)
C19	0.035 (2)	0.042 (2)	0.044 (3)	0.0041 (18)	0.0027 (19)	−0.0119 (19)
C22	0.051 (3)	0.048 (3)	0.068 (3)	−0.017 (2)	0.009 (2)	−0.001 (2)
C3	0.057 (3)	0.081 (4)	0.047 (3)	0.005 (3)	0.033 (2)	0.000 (2)
C4	0.071 (3)	0.074 (3)	0.046 (3)	0.007 (3)	0.031 (3)	−0.013 (2)
C18	0.040 (2)	0.057 (3)	0.031 (2)	0.010 (2)	0.0042 (19)	−0.009 (2)
C5	0.053 (3)	0.049 (3)	0.035 (2)	0.005 (2)	0.017 (2)	−0.0049 (19)
C1	0.043 (3)	0.112 (4)	0.054 (3)	−0.007 (3)	0.025 (2)	0.016 (3)
C10	0.036 (2)	0.059 (3)	0.038 (2)	0.0092 (19)	0.0068 (19)	0.0102 (19)
C12	0.041 (2)	0.081 (3)	0.046 (3)	0.000 (2)	0.020 (2)	0.000 (2)
C11	0.036 (2)	0.089 (3)	0.049 (3)	0.009 (2)	0.013 (2)	0.007 (2)
C13	0.041 (2)	0.055 (3)	0.041 (3)	−0.016 (2)	0.014 (2)	0.0015 (19)
O5	0.083 (3)	0.152 (4)	0.093 (3)	−0.045 (3)	0.045 (2)	−0.037 (3)

### Geometric parameters (Å, º)

**Table:**

Cu1—O3	1.900 (2)	C9—H9A	0.9800
Cu1—O2	1.912 (2)	C6—C5	1.409 (5)
Cu1—N1	1.928 (3)	C2—C3	1.366 (5)
Cu1—N2	1.961 (3)	C19—C18	1.393 (5)
N2—C15	1.280 (4)	C19—H19A	0.9300
N2—C14	1.490 (4)	C22—H22A	0.9600
O3—C21	1.317 (4)	C22—H22B	0.9600
O2—C7	1.308 (4)	C22—H22C	0.9600
N1—C8	1.292 (4)	C3—C4	1.398 (6)
N1—C9	1.472 (4)	C3—H3A	0.9300
C15—C16	1.438 (5)	C4—C5	1.353 (5)
C15—H15A	0.9300	C4—H4A	0.9300
C21—C16	1.413 (5)	C18—H18A	0.9300
C21—C20	1.436 (5)	C5—H5A	0.9300
C8—C6	1.439 (5)	C1—H1A	0.9600
C8—H8A	0.9300	C1—H1B	0.9600
O4—C20	1.367 (4)	C1—H1C	0.9600
O4—C22	1.436 (4)	C10—C11	1.532 (5)
C7—C6	1.413 (5)	C10—H10A	0.9700
C7—C2	1.433 (5)	C10—H10B	0.9700
C17—C18	1.361 (5)	C12—C13	1.510 (5)
C17—C16	1.411 (5)	C12—C11	1.513 (6)
C17—H17A	0.9300	C12—H12A	0.9700
C14—C13	1.527 (5)	C12—H12B	0.9700
C14—C9	1.534 (5)	C11—H11A	0.9700
C14—H14A	0.9800	C11—H11B	0.9700
C20—C19	1.375 (5)	C13—H13A	0.9700
O1—C2	1.373 (5)	C13—H13B	0.9700
O1—C1	1.422 (4)	O5—HW1	0.8458
C9—C10	1.511 (5)	O5—HW2	0.8437
			
O3—Cu1—O2	90.74 (10)	O1—C2—C7	113.5 (3)
O3—Cu1—N1	169.18 (11)	C20—C19—C18	121.0 (4)
O2—Cu1—N1	92.53 (11)	C20—C19—H19A	119.5
O3—Cu1—N2	93.78 (11)	C18—C19—H19A	119.5
O2—Cu1—N2	172.77 (11)	O4—C22—H22A	109.5
N1—Cu1—N2	84.05 (12)	O4—C22—H22B	109.5
C15—N2—C14	122.4 (3)	H22A—C22—H22B	109.5
C15—N2—Cu1	124.6 (2)	O4—C22—H22C	109.5
C14—N2—Cu1	112.1 (2)	H22A—C22—H22C	109.5
C21—O3—Cu1	126.5 (2)	H22B—C22—H22C	109.5
C7—O2—Cu1	124.5 (2)	C2—C3—C4	121.0 (4)
C8—N1—C9	120.5 (3)	C2—C3—H3A	119.5
C8—N1—Cu1	126.4 (2)	C4—C3—H3A	119.5
C9—N1—Cu1	112.9 (2)	C5—C4—C3	119.3 (4)
N2—C15—C16	126.2 (3)	C5—C4—H4A	120.3
N2—C15—H15A	116.9	C3—C4—H4A	120.3
C16—C15—H15A	116.9	C17—C18—C19	119.5 (4)
O3—C21—C16	125.4 (3)	C17—C18—H18A	120.2
O3—C21—C20	117.2 (3)	C19—C18—H18A	120.2
C16—C21—C20	117.4 (3)	C4—C5—C6	121.3 (4)
N1—C8—C6	124.5 (3)	C4—C5—H5A	119.3
N1—C8—H8A	117.8	C6—C5—H5A	119.3
C6—C8—H8A	117.8	O1—C1—H1A	109.5
C20—O4—C22	117.5 (3)	O1—C1—H1B	109.5
O2—C7—C6	125.4 (3)	H1A—C1—H1B	109.5
O2—C7—C2	118.5 (3)	O1—C1—H1C	109.5
C6—C7—C2	116.1 (3)	H1A—C1—H1C	109.5
C18—C17—C16	121.6 (4)	H1B—C1—H1C	109.5
C18—C17—H17A	119.2	C9—C10—C11	111.1 (3)
C16—C17—H17A	119.2	C9—C10—H10A	109.4
N2—C14—C13	116.3 (3)	C11—C10—H10A	109.4
N2—C14—C9	106.6 (3)	C9—C10—H10B	109.4
C13—C14—C9	111.1 (3)	C11—C10—H10B	109.4
N2—C14—H14A	107.5	H10A—C10—H10B	108.0
C13—C14—H14A	107.5	C13—C12—C11	112.1 (4)
C9—C14—H14A	107.5	C13—C12—H12A	109.2
O4—C20—C19	126.2 (4)	C11—C12—H12A	109.2
O4—C20—C21	113.1 (3)	C13—C12—H12B	109.2
C19—C20—C21	120.7 (4)	C11—C12—H12B	109.2
C2—O1—C1	118.0 (3)	H12A—C12—H12B	107.9
N1—C9—C10	110.3 (3)	C12—C11—C10	110.6 (3)
N1—C9—C14	105.9 (3)	C12—C11—H11A	109.5
C10—C9—C14	112.3 (3)	C10—C11—H11A	109.5
N1—C9—H9A	109.4	C12—C11—H11B	109.5
C10—C9—H9A	109.4	C10—C11—H11B	109.5
C14—C9—H9A	109.4	H11A—C11—H11B	108.1
C5—C6—C7	120.7 (3)	C12—C13—C14	112.6 (3)
C5—C6—C8	117.2 (3)	C12—C13—H13A	109.1
C7—C6—C8	122.1 (3)	C14—C13—H13A	109.1
C17—C16—C21	119.7 (3)	C12—C13—H13B	109.1
C17—C16—C15	117.9 (3)	C14—C13—H13B	109.1
C21—C16—C15	122.4 (3)	H13A—C13—H13B	107.8
C3—C2—O1	125.0 (4)	HW1—O5—HW2	104.7
C3—C2—C7	121.5 (4)		

### Hydrogen-bond geometry (Å, º)

**Table:**

*D*—H···*A*	*D*—H	H···*A*	*D*···*A*	*D*—H···*A*
O5—H*W*1···O1^i^	0.85	2.24	2.971 (5)	146
O5—H*W*1···O2^i^	0.85	2.48	3.161 (4)	138
C8—H8*A*···O3^ii^	0.93	2.44	3.352 (4)	166
C9—H9*A*···O2^ii^	0.98	2.65	3.579 (5)	159

Symmetry codes: (i) *x*+1, *y*, *z*; (ii) −*x*+1/2, *y*+1/2, −*z*+1/2.
